# The impact of falls on activities of daily living in older adults: A retrospective cohort analysis

**DOI:** 10.1371/journal.pone.0294017

**Published:** 2024-01-03

**Authors:** Claire E. Adam, Annette L. Fitzpatrick, Cindy S. Leary, Sindana D. Ilango, Elizabeth A. Phelan, Erin O. Semmens

**Affiliations:** 1 School of Public and Community Health Sciences, University of Montana, Missoula, Montana, United States of America; 2 Center for Population Health Research, University of Montana, Missoula, Montana, United States of America; 3 Department of Family Medicine, University of Washington, Seattle, Washington, United States of America; 4 Department of Epidemiology, School of Public Health, University of Washington, Seattle, Washington, United States of America; 5 Division of Gerontology and Geriatric Medicine, School of Medicine, University of Washington, Seattle, Washington, United States of America; 6 Department of Health Systems and Population Health, School of Public Health, University of Washington, Seattle, Washington, United States of America; University of Bergen: Universitetet i Bergen, NORWAY

## Abstract

**Background:**

Falls contribute to impairments in activities of daily living (ADLs), resulting in significant declines in the quality of life, safety, and functioning of older adults. Understanding the magnitude and duration of the effect of falls on ADLs, as well as identifying the characteristics of older adults more likely to have post-fall ADL impairment is critical to inform fall prevention and post-fall intervention. The purpose of this study is to 1) Quantify the association between falls and post-fall ADL impairment and 2) Model trajectories of ADL impairment pre- and post-fall to estimate the long-term impact of falls and identify characteristics of older adults most likely to have impairment.

**Method:**

Study participants were from the Ginkgo Evaluation of Memory Study, a randomized controlled trial in older adults (age 75+) in the United States. Self-reported incident falls and ADL scores were ascertained every 6 months over a 7-year study period. We used Cox proportional hazards analyses (n = 2091) to quantify the association between falls and ADL impairment and latent class trajectory modeling (n = 748) to visualize trajectories of ADL impairment pre-and post-fall.

**Results:**

Falls reported in the previous 6 months were associated with impairment in ADLs (HR: 1.42; 95% CI 1.32, 1.52) in fully adjusted models. Based on trajectory modeling (n = 748), 19% (n = 139) of participants had increased, persistent ADL impairment after falling. Participants who were female, lived in a neighborhood with higher deprivation, or experienced polypharmacy were more likely to have ADL impairment post-fall.

**Conclusions:**

Falls are associated with increased ADL impairment, and this impairment can persist over time. It is crucial that all older adults, and particularly those at higher risk of post-fall ADL impairment have access to comprehensive fall risk assessment and evidence-based fall prevention interventions, to help mitigate the negative impacts on ADL function.

## 1. Introduction

While many falls are preventable [1], a large proportion (20–33%) of older adults nonetheless fall each year [2,3]. Data are widely collected on outcomes from falls such as death and injury [3–5]; however, the long-term impacts of falls on the functional ability of older adults is less understood. Falls may result in injury, pain, fear of falling, and decreased physical activity [2,3,6]. These outcomes may cause difficulty with Activities of Daily Living (ADLs), which are measures of self-care ability [7]. Difficulty in performing ADLs may result in dependence on other individuals and/or assistive devices. ADL impairment is a predictor of hospitalization and mortality [8] and has multiple other adverse consequences, including an increased risk of institutionalization [9]; poorer mental health [10]; and increased need for formal (paid) and informal care [11], which in turn affect quality of life, strain financial resources, and burden health systems [3,10]. Incident difficulty with ADL functioning portends a trajectory of increased need for personal care, nursing home placement, and heightened risk of death [10,12–14]. The ADLs included in this study are as follows: walking around the home, transferring from a chair or bed, eating, dressing, bathing, and toileting. Impairment in specific ADLs is associated with increased risk of declines in mental health (eating, bathing, dressing, toileting, transfers, and walking [10]), increased risk of nursing home admission (bathing and walking [13]), and decreased social interaction (walking [14]). Identifying modifiable risk factors, such as falls, may reduce the likelihood of these adverse outcomes. Quantifying the risk of ADL impairment post-fall is thus necessary to fully characterize the impact of falls on older adults, the healthcare system, and public health.

A limited number of studies have looked at the association between falls and impairment in performing ADLs. While most found an association between falls and increased impairment in ADLs, the type of fall (all, injurious, multiple) varied across studies, and many of the studies were cross-sectional or had a short follow-up time [15–25], making it challenging to characterize the range of post-fall trajectories and identify those more susceptible to poorer post-fall functioning. It is critical to examine both medically treated and non-medically treated falls, as the majority of falls older adults experience are not medically treated [3].

A comprehensive assessment of covariates as part of an analysis examining falls and their relationship to ADL function is also of fundamental importance. For example, in older adults, mild cognitive impairment (MCI) is prevalent and increases with age [26], older adults with MCI are a vulnerable population, and MCI is associated both with increased risk of falls [27] and increased risk of ADL impairment [28–30]. However, no studies were identified that specifically looked at the association between falls and ADL impairment in older adults with MCI. As another example, higher neighborhood deprivation is associated with increased fall risk [2,31,32] and ADL impairment[33,34]. Evidence of an increased risk of long-term ADL impairment post-fall for people experiencing higher neighborhood deprivation would strengthen the argument for prioritizing funding and access to fall prevention programs in neighborhoods with lower socioeconomic status. Hospitalization [35,36] and polypharmacy [37,38] are associated with both an increased risk of falls and ADL impairment, and are therefore critical to include in an analysis of the impact of falls on ADLs.

The objectives of this study are to: 1) Quantify the association between falls and post-fall ADL impairment and 2) Model trajectories of ADL impairment pre- and post-fall to estimate the long-term impact of falls and identify characteristics of older adults most likely to have impairment. Trajectory modeling allows for a more thorough understanding of the common paths post-fall with respect to ADL impairment, both in terms of the duration of post-fall impairment and heterogenous and potentially non-linear trajectories. Previous findings of an association between falls and ADL impairment are important in establishing a history of falls as a risk factor for ADL impairment; the present study seeks to further characterize the association, by differentiating between older adults who do and do not experience ADL impairment after a fall, and identifying the characteristics of those most at risk. It is crucial that all older adults, and particularly those at higher risk of post-fall ADL impairment have access to comprehensive fall risk assessment and evidence-based fall prevention interventions, to help mitigate the negative impacts on ADLs. We hypothesized that older adults who fell would have more ADL impairment post-fall than older adults who had not fallen, and that this impairment would persist over time. Additionally, older adults who fell would have heterogenous trajectories of ADL impairment pre- and post- fall, with some older adults at greater risk of worse functioning post-fall based on demographic, health, and social characteristics, specifically those with cognitive impairment and those experiencing higher levels of neighborhood deprivation.

## 2 Materials and methods

### 2.1 Study population

This study utilized data from the Ginkgo Evaluation of Memory Study (GEMS). Detailed descriptions of study methodology have been published [39,40]. Briefly, GEMS was a randomized controlled trial that investigated whether *Ginkgo biloba* supplementation decreased the risk of dementia or cognitive decline in older adults [41,42]. *Ginkgo biloba* had no effect on dementia or cognitive decline [41,42]. GEMS participants were recruited from September 2000 to June 2002 [41]. GEMS follow-up occurred from 2000 to 2008 and included 3,069 older adults, who were community-dwelling at study-entry. There were four study sites: Sacramento, CA, Pittsburgh, PA, Hagerstown, MD, and Winston-Salem and Greensboro, NC. Participants were eligible for participation in GEMS if they were free of dementia at baseline, not taking anticoagulants, certain antidepressants, medications for cognition, *Ginkgo biloba*, or high doses of vitamin E, did not have Parkinson’s disease, specific blood tests were within the normal range, and their life-expectancy was greater than 5 years [41]. Participants were followed for a median of 6.1 years [41]. While enrolled, participants had study visits every 6 months. The University of Montana Institutional Review Board for the Protection of Human Subjects in Research approved the study described here through written consent. The study was determined to be secondary data analysis and not human subjects research. All analyses for this study were conducted using deidentified data, and the authors completing the analyses did not have access to data with identifiers. Data were accessed for these analyses in May 2021.

### 2.2 Fall ascertainment

Participants were asked at each 6-month study visit, beginning at the one-year study visit “In the past six months since we last saw you, have you had a fall?”. Participants could respond, “yes,” “no,” or “don’t know.” If they responded “yes,” they were then asked how many times they had fallen in the past six months” and if any fall required medical treatment, to which they could respond “yes,” “no,” or “don’t know.” Falls were dichotomized as “yes” or “no” for a fall in the previous 6 months. Any response of “don’t know” was coded as missing. Previous falls were included as a continuous covariate and included all falls that had occurred up to the current study visit. Participants were excluded from analyses if they did not have any fall reporting periods during the study.

### 2.3 Activities of daily living

The outcome of interest was the score on the GEMS Activities of Daily Life (ADL) questionnaire (Table 1). GEMS ADL items map to other commonly used measure of ADL functioning and have been used in prior published analyses of this outcome [40,41,43]. Responses about selected ADLs from the questionnaire were included in analyses (Table 1). Participants answered the ADL questionnaire at the screening visit and then every 6 months starting at the one-year visit until the four-year visit, and then annually for the rest of the study for a total of up to 10 measures. Participants were asked (yes/no) whether they had any difficulty performing each ADL, (Table 1). Each “yes” response, indicating difficulty with performing the ADL, was scored as one, while each “no” or “could do it but don’t for reason other than health” was scored as zero, and “don’t know” or refused to answer, was coded as missing. For some items on the questionnaire, participants were asked if they had difficulty with this ADL, whereas for other items, they were additionally asked if they were unable to perform this ADL (Table 1). Scores were incorporated into analyses as both a dichotomous (i.e., no impairment/impairment) and a continuous outcome, the total number of ADLs a participant reported having impairment with. “No impairment” was defined as having a score of 0; “impairment” was defined as having a score of one [12,30,44,45].

**Table 1:** ADLs included from the GEMS activities of daily life questionnaire*.

1. Walking around your home^a^
2. Getting out of a bed or a chair^a^
3. Eating, including feeding yourself^b^
4. Dressing yourself^b^
5. Bathing or showering^b^
6. Using the toilet, including getting to the toilet^b^

*Modified from GEMS study forms.

^a^For these items, participants were asked: Do you have any difficulty?.

^b^For these items, participants were asked: Because of health or physical problems, do you have any difficulty, or are you unable to?.

Response options: Yes, no, could do it, but don’t for reason other than health, don’t know or refused.

### 2.4 Covariates

#### 2.4.1 Cognition

Impaired cognition is associated with increased risk of falling [27,46,47], and decreased ability to perform ADLs [25,28]. Cognition was assessed at multiple visits during the study period and was included as a time-varying covariate as intact cognition, MCI, and dementia.

MCI was ascertained at baseline, if a participant failed the dementia screening at any study visit, and also annually beginning in 2004. Participants who were impaired on two or more tests in the neuropsychological battery, based on cut-points from participants in the Cardiovascular Health Study, and scored 0.5 on the Clinical Dementia Rating Scale, were determined to have MCI [39]. The criteria for MCI were based on the 2004 International Working Group on Mild Cognitive Impairment guidelines [48]. Participant’s cognitive status at baseline was carried forward from baseline until annual assessments began unless they were diagnosed with MCI or dementia in the interim.

GEMS participants were screened for dementia at each study visit, and those who did not pass two of the three screening tests went through a full neuropsychological battery, comprised of ten tests covering five cognitive domains, and based on those results were referred for a full neurological exam and MRI if dementia was suspected [41]. Those who were diagnosed with dementia ended their participation in GEMS at diagnosis. The focus of the current research was on intact cognition and MCI; however, dementia was included as a third cognitive category as some participants had data from the study visit(s) during the time when dementia was first suspected due to failed screening tests and when all study protocols were completed for a dementia diagnosis.

#### 2.4.2 Additional covariates

Sex, age, study site, and whether participants took *Gingko biloba* or placebo were selected *a priori* to be included in the analyses. Other covariates considered for inclusion in the models include neighborhood deprivation index, medication use, hospitalization in the previous six months, and education. Neighborhood deprivation index (NDI) characterizes neighborhoods at the census tract level [49,50]. NDI is a weighted linear combination of percentage of people within a census tract with a bachelor’s degree, in managerial occupations, with at least a high school education, with an annual household income greater than $50,000, and the percent interest, dividend, or rental income, median home value, and median household income [49,50]. A higher NDI score indicates more neighborhood deprivation. Higher NDI is associated with both increased risk of falls [2,31,32] and decreased ability to perform ADLs [33,34]. NDI was included as a time-varying covariate. Education was assessed at the baseline visit, whereas medication use and hospitalization were assessed at each 6-month study visit. Education was a categorical variable; high school education or less, college, and greater than four years of college. Education was assessed as a covariate, as there is evidence of an inverse association between a higher level of education and risk of ADL impairment [51] and risk of falls [52]. Medication use was included as the number of prescription medications a participant was taking at each study visit and was categorized as no polypharmacy (0–4 medications) or polypharmacy (5+ medications) [37,38]. Polypharmacy is associated both with increased fall risk [37] and increased risk of ADL impairment [53]. Participants reported hospitalizations for the previous 6 months, with a hospitalization defined as at least a one-night stay in the hospital for any reason. The variable was dichotomized as yes/no for any hospitalization in the previous 6 months.

### 2.5 Statistical approach

To estimate the association between falls and post-fall ADL impairment, Cox proportional hazards models were used with age as the time scale. Because of potential zero-inflation for the scores for ADLs (79% zeros) and interest in understanding the risk of any ADL impairment, we dichotomized the outcome to ADL impairment/no impairment and used Cox proportional hazards models for recurrent events, for any fall that occurred for a participant during the study period [54–56]. Sex (categorical, time independent), NDI (continuous and categorical-quartiles, time-varying), and cognition (categorical- intact, MCI, and dementia, time-varying) were chosen *a priori* for their known association both with falls and ADLs [2,24,28,32,33,46]. Study site (categorical, time-independent) and whether participants received *G*. *biloba* or placebo (categorical, time independent) were also included as covariates in the model to adjust for any potential impacts of the study design. Polypharmacy (categorical, time-varying), hospitalization in the previous 6 months (categorical, time-varying), and education (categorical, time-independent) were all considered for inclusion during model building. Previous number of falls (continuous, time-varying) was also included in the models. Schoenfeld residuals were used to assess the proportional hazards assumption. All analyses were completed using R statistical software.

We described the heterogeneous trajectories of ADL impairment post-fall using latent class trajectory modeling. Latent class trajectory modeling has been used to characterize trajectories of disability post serious fall injury [21]. We used latent class trajectory modeling to describe and visualize the severity and duration of ADL impairment post-fall, and the participant characteristics associated with these trajectories, increasing understanding of which populations are at highest risk post-fall. ADL trajectories were modeled for participants who did not fall during the study period and had at least three ascertainments of ADL scores as the comparison group, with age as the time axis. ADL trajectories, pre-and post-fall, were modeled only for those participants who reported an incident fall during the study and had at least three pre- and post-incident fall ADL measures [57,58]. Pre-fall time was considered to start at the first study visit, and the first ascertainment of falls occurred at the one-year study visit. Study visits were approximately six months apart, and timing of the pre- and post-fall ADL measurements were rounded to the nearest six-month time period. Participants who had study visits closer together than 6 months apart (mean = 181 days, min = 89 days, max = 761 days), contributed more than one ADL score for that specific 6-month period. The process of trajectory modelling included determining whether to include random effects, checking the shape of the model (quadratic, linear, splines), and choosing the number of classes (trajectories) for each model. Log-likelihood, Akaike information criterion (AIC), model convergence, having at least 1% of the study population in each class, and having a mean posterior probability for each class of greater than 70%, were the criteria used to choose the number of classes in the final models [57,59,60]. Participant characteristics associated with each latent class were reported for each model. We used t-tests, test of proportions, or Chi-squared tests, as appropriate, to compare the characteristics of participants in each pre-fall and post-fall trajectory. Additionally, we performed logistic regression to quantify the odds of post-fall latent class membership for each characteristic of interest (age, gender, MCI, education, NDI, polypharmacy, hospitalization, total falls, and pre-fall latent class). These analyses were adjusted for the study design treatment assignment and clinic site. All analyses were completed using the *lcmm* function in the *lcmm* package in R statistical software, which was selected to account for the non-gaussian distribution of ADL scores and repeated measures of ADL scores [58,60].

## 3. Results

### 3.1 Participant characteristics

The Cox proportional hazards models included 2901 participants (Table 2). Participants were excluded for missingness sequentially, for falls and number of falls (n = 129), NDI (n = 17), and missing visits (n = 22).

**Table 2:** Baseline participant characteristics by fall status (n = 2901).

Characteristic	All n = 2901 n or mean (% or SD)	No Falls n = 796 n or mean (% or SD)	At least one Fall n = 2105 n or mean (% or SD)
Age	78.6 (3.3)	78.4 (3.1)	78.7 (3.3)
Sex (Female)	1336 (46%)	340 (43%)	996 (47%)
Race- White	2774 (96%)	755 (95%)	2019 (96%)
Treatment (Ginkgo, yes)	1464 (51%)	398 (50%)	1066 (51%)
Education -Highschool or less	1035 (36%)	292 (37%)	743 (35%)
Mild cognitive impairment (yes)	464 (16%)	141 (17%)	323 (15%)

More participants had a fall 73% (n = 2105) than did not 27% (n = 796) during the study period. Those who fell were more likely to be female, and to be older at baseline (p-value <0.05, test of proportions, and t-test) (Table 2). The majority of falls (75%) during the study period were not medically treated.

### 3.2 The association between falls and ADL impairment

At baseline, 17% (n = 505) of participants had ADL impairment. This increased to 25% (n = 725) for participants at their last observed study visit. The ADLs that the largest percent of participants reported impairment in, both at baseline and the last observed visit, were transferring to/from a bed or chair (16% (n = 437) at baseline, 18% (n = 514) at last observed visit), followed by walking around the home (5% (n = 136), at baseline, 9% (n = 258) at last observed visit).

Participants included in the Cox proportional hazards models analyses had a median of seven (minimum = 1, maximum = 10) study visits with data on falls, ADLs, and included covariates. One or more falls in the 6-month fall reporting period was significantly associated with a 42% higher (95% CI: 1.32 to 1.52) risk of post-fall ADL impairment in a model adjusted for sex, study site, study treatment, cognition, polypharmacy, NDI, hospitalization, and previous number of falls.

### 3.3 Trajectories of ADL impairment

**Fig 1:**
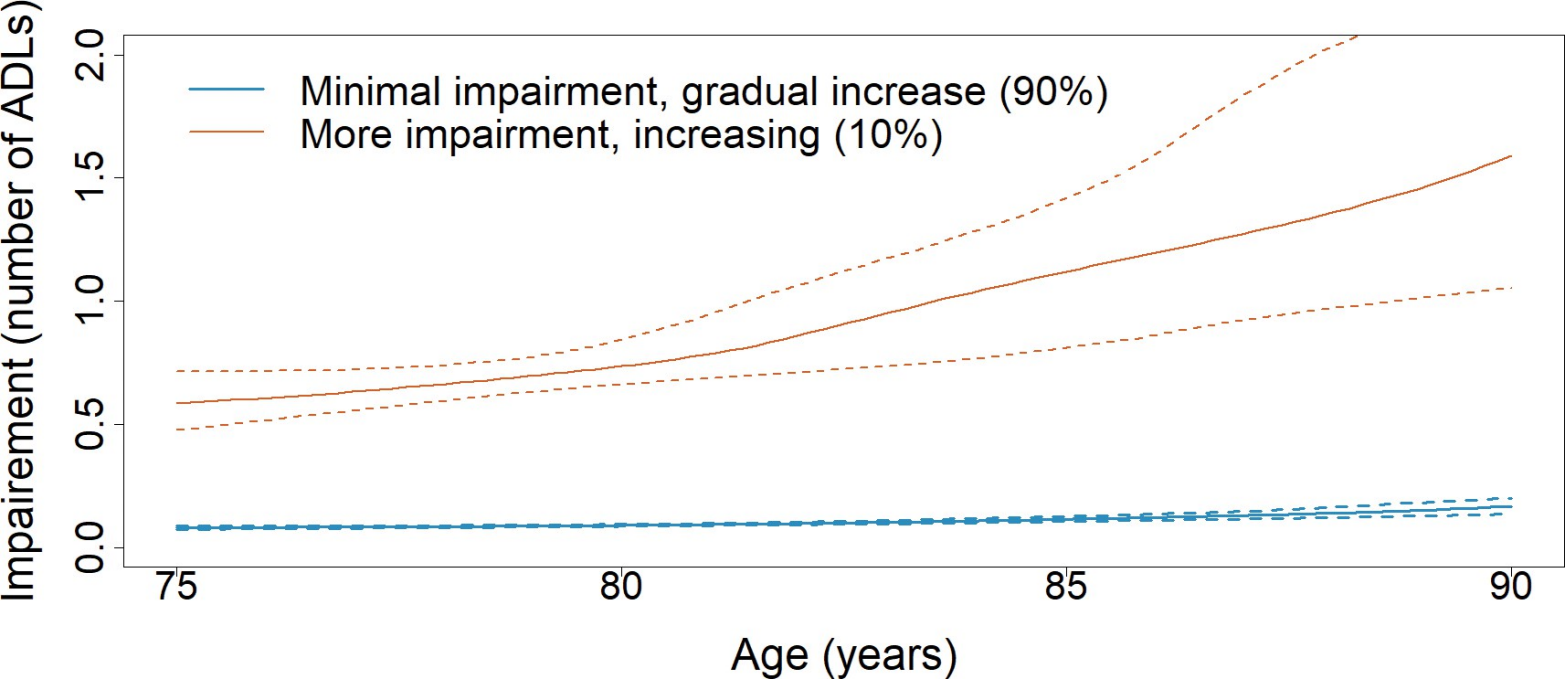
Trajectories of ADL impairment for participants who did not fall during the study period (n = 762). Trajectories of ADL impairment with 95% confidence intervals (dotted lines).

Trajectory models for participants who did not fall during the study period included participants who had at least three measurements (n = 762; median = 8, min = 3, max = 11) of ADL scores. Visualization of trajectories (Fig 1) were restricted to the age range of 75 to 90 years old to ensure ascertainment of ADL scores for at least 5% of the included participants at each age. Trajectory models for participants who did not fall were included for comparison.

For participants who did not fall, two latent classes were identified for ADL impairment; one class with minimal impairment that gradually increased with age (90%, n = 687), and one class with more impairment that continued to increase with age (10%, n = 75) (Fig 1). The trajectory models included random effects for slope and intercept, and splines. Posterior probabilities were above 96% for both classes.

**Fig 2:**
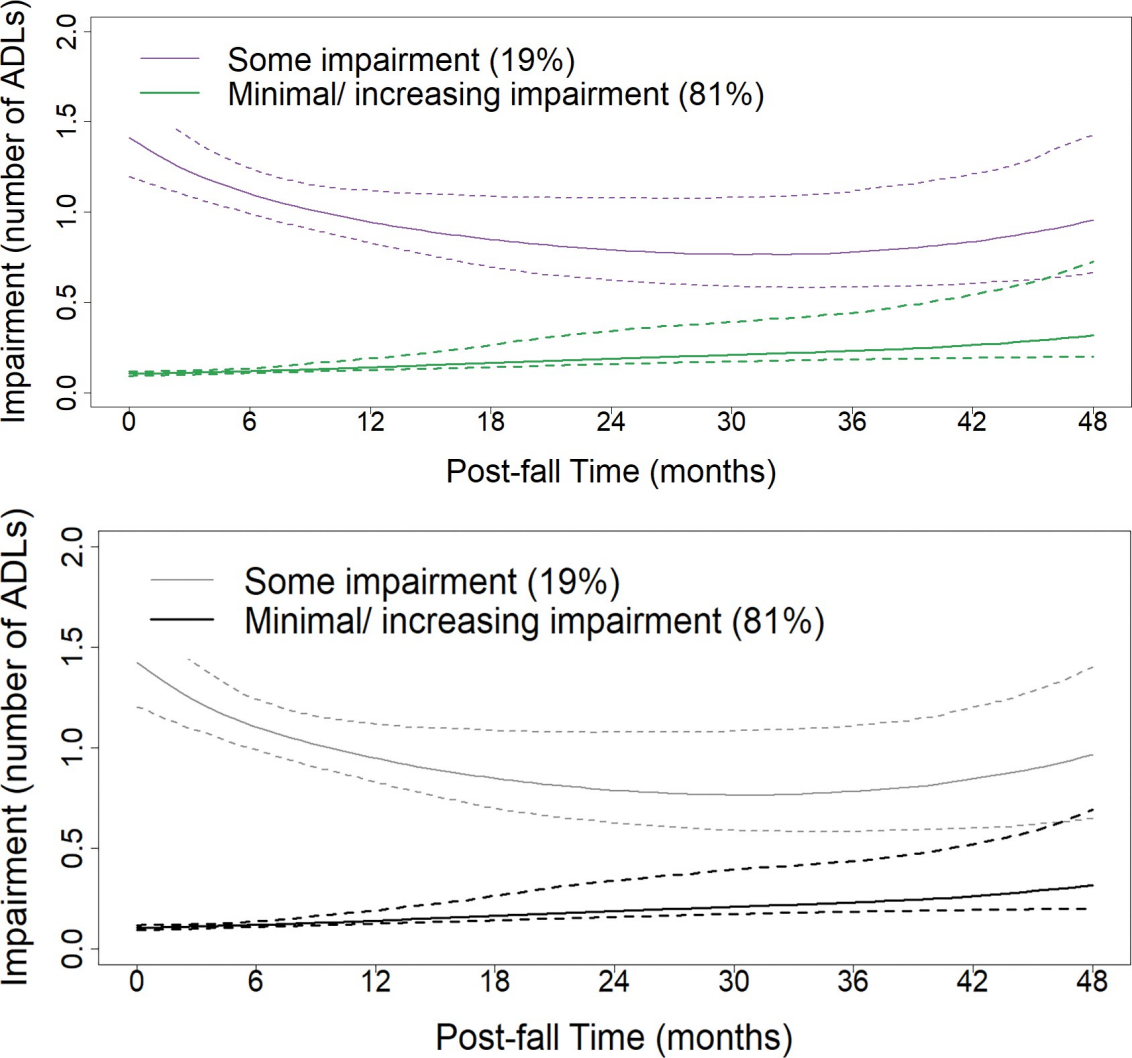
Pre- and post- fall trajectories of ADL impairment (n = 748). a. Pre-fall. b. Post-fall. (A and B) Trajectories of ADL impairment with 95% confidence intervals (dotted lines).

Models for participants who fell included participants (n = 748) with a fall during the study period and at least three ADL scores both pre- and post-fall [57,58]. We modeled ADL scores pre- and post- first incident fall during the study (Fig 2A and 2B). The median number of ADL measures pre-fall was four (min = 3, max = 8). Post-fall, the median number of ADL observations was four (min = 3, max = 7). Both pre-fall time (Fig 2A, 42 to 6 months prior to a fall) and post-fall time (Fig 2B, 0 to 48 months post-fall) were restricted to include ADL scores from at least 5% of participants at each 6 month-interval.

Trajectory models for ADL impairment pre-fall included two latent classes (Fig 2A). The model included a quadratic term for time, splines, and random effects for intercept and slope. Pre-fall, 85% (n = 636) of participants were in a class with minimal to no ADL impairment, and 15% (n = 112) were in a class characterized by some ADL impairment. Post-fall, two latent classes were identified, with 19% (n = 139) of participants in a class with increased impairment post-fall that gradually decreased over time, and 81% (n = 609) in a trajectory with minimal impairment with a slight increase over time (Fig 2A). A greater proportion of participants were in a trajectory of more ADL impairment post-fall (19%, n = 139) than pre-fall (15%, n = 112). The pre- and post-fall classes met the criteria of greater than 1% of participants in each class [57]. The mean posterior probabilities for each pre-fall class were above 93%, and above 92% for the two post-fall classes, both exceeding the criterium of 70% [57].

Participants in the pre-fall class with some impairment, were more likely to be older, have MCI, live in a neighborhood with more deprivation, have a high school education or less, and have polypharmacy (Table 3).

**Table 3:** Participant characteristics by latent class for trajectories of ADL impairment pre-and post-fall (n = 748).

		Pre-fall		Post-fall	
	All (baseline) n = 748 (100%)	Some impairment n = 112 (15%)	Minimal/ no impairment n = 636 (85%)	Some impairment n = 139 (19%)	Minimal/ increasing impairment n = 609 (81%)
**Characteristics**					
Age	78.6 (3.3)	81.6 (3.7)*	80.7 (3.2)*	82.0 (4.0)	81.4 (3.1)
Sex (female)	330 (44%)	57 (51%)	273 (43%)	73 (53%)*	257 (42%)*
Ginkgo biloba (yes)	362 (48%)	54 (48%)	308 (48%)	67 (48%)	295 (48%)
Education (high school or less)	266 (36%)	49 (44%)*	217 (34%)*	48 (35%)	218 (36%)
MCI (yes)	85 (11%)	22 (20%)*	64 (10%)*	24 (17%)	83 (14%)
NDI quartiles^a^		*	*	*	*
1	177 (24%)	17 (15%)	163 (26%)	21 (15%)	161 (27%)
2	199 (27%)	29 (26%)	172 (27%)	43 (31%)	154 (25%)
3	189 (25%)	25 (22%)	161 (25%)	30 (22%)	159 (26%)
4	180 (24%)	41 (37%)	137 (22%)	45 (32%)	132 (22%)
Polypharmacy (yes)	224 (30%)	50 (45%)*	204 (32%)*	69 (50%)*	209 (34%)*
Hospitalization (yes)	65 (9%)	13 (12%)	42 (7%)	24 (17%)	72 (12%)
Total number of falls	NA	NA	NA	4.3 (4.7)*	3.5 (5.8)*
Pre-fall Category*					
Some Impairment	NA	112 (100%)	0 (0%)	64 (57%)	48 (43%)
Minimal/ no impairment	NA	0 (0%)	636 (100%)	75 (12%)	561 (88%)

*Note*: Characteristics were measured at baseline visit, and the year-one visit (polypharmacy and hospitalization) for all participants, the visit prior to falling for pre-fall characteristics, at the visit after the incident fall for post-fall characteristics, and for total number of falls at the last observed visit. Abbreviations: MCI (Mild cognitive Impairment), NDI (Neighborhood Deprivation Index, 1 is least deprivation, 4 is most), NA (not applicable).

^a^Missing values for NDI (n = 3)

*p-value < = 0.05, t-test, test of proportions, or Chi-squared test.

Post-fall, 19% (n = 139) of participants experienced increased ADL impairment (Fig 2B). The magnitude of the impairment decreased over time; however, it remained greater over time than for the 81% (n = 609) who fell and were in the minimal/ increasing impairment trajectory (Fig 2B). Post-fall, participants with increased ADL impairment were more likely to be female (OR: 1.53, 95% CI: (1.05 to 2.22), live in a neighborhood with the most deprivation, (OR: 2.16, 95% CI: (1.21 to 3.96), and have polypharmacy (OR: 1.91, 95% CI: (1.31 to 2.79) (Tables 3 and S1). They also experienced a greater mean number of falls (4.3 compared to 3.5) during the study period (Table 3). Participants who had some impairment pre-fall had a greater probability of having some impairment post-fall (OR: 9.46, 95% CI 6.06 to 14.90).

## 4. Discussion

This study found that older adults who experience falls have a significantly heightened risk, 42% greater, of post-fall ADL impairment, and for many older adults, this impairment persists for a prolonged period of time (i.e., years). The trajectory models in conjunction with the Cox proportional hazards models provide evidence of increased risk of greater, sustained ADL impairment after a fall. Certain older adults are more likely to have increased impairment post-fall. These include females, those residing in a neighborhood with more deprivation, and those with polypharmacy.

Our study findings for the association between falls and ADL impairment are of a similar magnitude to one study [16] and slightly lower than two studies [18,22], both of which had study populations outside of the United States and used a cross-sectional study design. Our study benefited from repeated measures of falls and ADL scores in addition to robust determination and inclusion of cognition as a covariate.

Based on trajectory models for those participants who fell, about 19% (n = 139) of participants had increased ADL impairment post-incident fall. This impairment appeared to decrease over about a two-year time period, and then remained fairly constant. The magnitude of impairment was greater than for the other 81% (n = 609) of participants who fell, until about four years post-incident fall. These trajectory models provide evidence that while ADL impairment increases as age increases (Fig 1), the proportion of older adults who develop ADL impairment (19%) is nearly double for those who fall, than those who do not fall (10%). While some participants had some impairment pre-fall, both the proportion of participants and the magnitude of impairment increased post-fall. A study looking at trajectories after a serious fall injury found that 64% of participants had little to no improvement in ADLs 12 months post-fall injury [21]. Those results in conjunction with the results of the present study add to the evidence that falls can have a long-lasting impact on older adults’ ability to perform basic self-care activities that are essential for independent living–a key goal of most if not all older adults [61].

Our study extended the literature on the relationship of falls to ADL impairment by shedding light on characteristics of those more likely to experience ADL impairment post-fall. These include females, those living in neighborhoods with higher deprivation, and those with polypharmacy. Post-fall, females were more likely to have ADL impairment. There is evidence that women are both more likely to fall [2] and to have ADL impairment [15,16]. Polypharmacy may be an indicator of more comorbidities, which could negatively impact older adult’s ability to perform ADLs [38], in addition to being an increased risk factor for falls [37]. There is evidence of an increased risk of disability for those living in neighborhoods with higher level of deprivation, following a critical illness [62]. Lack of access to medical care may contribute to the increased risk of ADL impairment post-fall for older adults in neighborhoods with more deprivation, as medical care is recommended to address post-fall healthcare needs, including prevention of future falls [63,64]; and higher levels of neighborhood deprivation are associated with reduced access to medical services [65]. Pre-fall, participants with MCI were more likely to have greater ADL impairment; however, while post-fall this trend continued, the difference was not statistically significant. This result was somewhat surprising as MCI is associated with increased risk of ADL impairment [28,29]. Study participants with MCI may not have had an increased likelihood of having greater ADL impairment post-fall, the sample size of participants may have been too small to detect a statistically significant difference, or this result could be due to recall bias or misclassification of MCI.

The characteristics of those participants at higher risk of negative outcomes post-fall range from non-modifiable (sex) to potentially modifiable (polypharmacy and multiple falls) [64]. For those characteristics that are not modifiable (sex) or less modifiable (NDI), ensuring that fall prevention is accessible to these subgroups of older adults is an essential component of reducing the impact of post-fall functional decline. With potentially modifiable characteristics, such as polypharmacy and multiple falls, these results provide further support for comprehensive fall risk assessment including medication use review [66,67]. Identifying individuals who may be more at-risk of ADL impairment post-fall, based on these characteristics, can help medical professionals, community organizations, and public health professionals provide targeted interventions to mitigate loss of function post-fall. Comprehensive fall risk assessment and evidence-based fall prevention interventions specifically designed for and accessible to these subgroups of older adults have the potential to reduce both the prevalence and magnitude of ADL impairment experienced by older adults, and further research is needed in this area.

This study had multiple strengths. The study population was relatively large, and the study was longer in duration, and had more measures than other studies identified that assessed the association between falls and ADLs. It was the only study found to include participants with MCI, a group at high risk of falls [27] and ADL impairment [28,29]. NDI was included in this study, a variable that is associated with both increased fall risk and ADL impairment [32–34]. The design of GEMs also allowed for the inclusion of multiple covariates of interest including polypharmacy and prior hospitalizations, both of which address the health status of participants, important risk factors for falls [2,3,37,68] and ADL impairment [38,69]. The percentage of participants with ADL impairment at baseline is similar to that of a large scale study done in the United States with a comparable mean age of participants [8].

Our analysis was limited by missing data on falls, and the study would have benefitted from more frequent measures of falls to reduce any potential recall bias, as retrospective reporting of falls is associated with underreporting of falls [70,71]. The generalizability of the study may be limited due to the characteristics of the study participants, who were highly educated, were excluded if taking certain common medications at baseline including anticoagulants and antidepressants, were predominantly White and were community dwelling at baseline. The frequency of measurement of MCI changed during the study period, potentially missing changes in cognitive status in the first half of the study, when it was measured only one time in about four years for most participants, compared to annually in the second half of the study. While trajectory modeling provided pertinent information about the impact of falls on the duration and level of ADL impairment, we did have to exclude a large number of study participants who fell, but did not have three ascertainments of ADL scores both pre- and post-fall, in order to follow best practice guidelines for trajectory modeling [57,58]. Those excluded were participants who fell early in the study, or towards the end of the study, and potentially those participants who left the study early due to a diagnosis of dementia, death, or loss to follow-up. Including these participants may have altered the pre- and post-fall trajectories we observed, for example, those who left the study early due to dementia may have had more ADL impairment, due to cognitive impairment [25,28].

## 5. Conclusions

We found that falls were associated with a 42% higher risk of ADL impairment. Post-fall, some study participants experienced persistent ADL impairment. We found that older adults who were female, lived in a neighborhood with higher deprivation, or had polypharmacy were more likely to have increased ADL impairment post-fall. Importantly, some of these characteristics, such as polypharmacy and multiple falls are potentially modifiable; whereas others, such as higher NDI, can be addressed by ensuring older adults in areas with higher NDI have access to both evidenced-based fall prevention and post-fall follow-up care. Falls put older adults at risk of ongoing ADL impairment. The magnitude and duration of ADL impairment further highlights the importance of fall prevention, and the importance of intervention for post-fall ADL impairment.

## Supporting information

S1 Table Associations between participant characteristics and membership in the post-fall trajectory class of “Some impairment” (n = 748). (DOCX)
