# Supplementary material for: The impact of falls on activities of daily living in older adults: A retrospective cohort analysis
Source: PLoS One. 2024 Jan 3;19(1):e0294017. doi: 10.1371/journal.pone.0294017 (PMC10763967; doi:10.1371/journal.pone.0294017)
Supplement: S1 Table — (DOCX) [file pone.0294017.s001.docx]

Table S1. Associations between participant characteristics and membership in the post-fall trajectory class of “Some impairment” (n=748).

| **Variable** | **Odds Ratio** | **95% confidence interval** | **Standard error** | **p-value** |
| --- | --- | --- | --- | --- |
| Age (continuous) | 1.05 | 1.00 to 1.11 | 0.03 | 0.057 |
| Female gender | 1.53 | 1.05 to 2.22 | 0.19 | 0.025 |
| MCI (yes) | 1.52 | 0.89 to 2.50 | 0.26 | 0.112 |
| Education (reference Highschool or less) |  |  |  |  |
| Some college | 1.23 | 0.80 to 1.90 | 0.22 | 0.350 |
| College graduate or more | 1.07 | 0.64 to 1.78 | 0.26 | 0.790 |
| NDI quartiles (reference, least deprivation)^a^ |  |  |  |  |
| 2 | 1.93 | 1.09 to 3.48 | 0.29 | 0.026 |
| 3 | 1.20 | 0.64 to 2.26 | 0.32 | 0.571 |
| 4 (most deprivation) | 2.16 | 1.21 to 3.96 | 0.30 | 0.010 |
| Polypharmacy (yes) | 1.91 | 1.31 to 2.79 | 0.19 | <0.001 |
| Hospitalization | 1.58 | 0.93 to 2.60 | 0.26 | 0.082 |
| Total Falls (continuous)^a^ | 1.02 | 0.99 to 1.05 | 0.02 | 0.206 |
| Pre-fall category -Some impairment | 9.46 | 6.06 to 14.90 | 0.23 | <0.001 |

*Note:* All logistic regression models adjusted for clinic site and treatment (Ginkgo (yes/no). Characteristics were measured at the visit after the incident fall for post-fall except for total number of falls, which was measured at the last observed visit. Abbreviations: MCI (Mild cognitive Impairment), NDI (Neighborhood Deprivation Index: 1 is least deprivation, 4 is most)

^a^Missing values for NDI (n=3) and total falls (n=3)
